# Effects of Germline CYP2W1*6 and CYP2B6*6 Single Nucleotide Polymorphisms on Mitotane Treatment in Adrenocortical Carcinoma: A Multicenter ENSAT Study

**DOI:** 10.3390/cancers12020359

**Published:** 2020-02-04

**Authors:** Barbara Altieri, Silviu Sbiera, Sabine Herterich, Silvia De Francia, Silvia Della Casa, Anna Calabrese, Alfredo Pontecorvi, Marcus Quinkler, Tina Kienitz, Massimo Mannelli, Letizia Canu, Anna Angelousi, Vasileios Chortis, Matthias Kroiss, Massimo Terzolo, Martin Fassnacht, Cristina L. Ronchi

**Affiliations:** 1 Division of Endocrinology and Diabetes, Department of Internal Medicine I, University Hospital, University of Würzburg, 97080 Würzburg, Germany; Altieri_B@ukw.de (B.A.); sbiera_S@ukw.de (S.S.); kroiss_M@ukw.de (M.K.); Fassnacht_M@ukw.de (M.F.); 2 Division of Endocrinology and Metabolic Diseases, Catholic University of the Sacred Heart, 00168 Rome, Italy; Silvia.DellaCasa@unicatt.it (S.D.C.); alfredo.pontecorvi@unicatt.it (A.P.); 3 Central Laboratory, University Hospital of Würzburg, 97080 Würzburg, Germany; herterich_S@ukw.de; 4 Department of Clinical and Biological Sciences, University of Turin, San Luigi Gonzaga Hospital, 10043 Turin, Italy; silvia.defrancia@unito.it; 5 Division of Internal Medicine I, University of Turin, San Luigi Gonzaga Hospital, Orbassano, 10043 Turin, Italy; anna.calabrese678@gmail.com (A.C.); massimo.terzolo@unito.it (M.T.); 6 Endocrinology in Charlottenburg, 10627 Berlin, Germany; marcusquinkler@t-online.de (M.Q.);; 7 Department of Endocrinology, Diabetes and Nutrition, Charité-Universitätsmedizin Berlin, Campus Mitte, 10117 Berlin, Germany; 8 Department of Experimental and Clinical Biomedical Sciences “Mario Serio”, University of Florence, 50134 Florence, Italy; massimo.mannelli@unifi.it (M.M.); letizia.canu@unifi.it (L.C.); 9 1st Propaedeutic Department of Internal Medicine, National and Kapodistrian University of Athens, Laiko Hospital, 11527 Goudi, Greece; a.angelousi@gmail.com; 10 Institute of Metabolism and System Research, University of Birmingham, Birmingham B152TT, UK; V.Chortis@bham.ac.uk; 11 Centre for Endocrinology, Diabetes and Metabolism (CEDAM), Birmingham Health Partners, Birmingham B152TT, UK; 12 Comprehensive Cancer Center Mainfranken, University of Würzburg, 97080 Würzburg, Germany

**Keywords:** adrenocortical carcinoma, mitotane, CYP2W1, CYP2B6, SNP, biomarker, predictive marker

## Abstract

Mitotane is the only approved drug for advanced adrenocortical carcinoma (ACC) and no biomarkers are available to predict attainment of therapeutic plasma concentrations and clinical response. Aim of the study was to evaluate the suitability of cytochrome P450(CYP)2W1 and CYP2B6 single nucleotide polymorphisms (SNPs) as biomarkers. A multicenter cohort study including 182 ACC patients (F/M = 121/61) treated with mitotane monotherapy after radical resection (group A, *n* = 103) or in not completely resectable, recurrent or advanced disease (group B, *n* = 79) was performed. CYP2W1*2, CYP2W1*6, CYP2B6*6 and *CYP2B6 rs4803419* were genotyped in germline DNA. Mitotane blood levels were measured regularly. Response to therapy was evaluated as time to progression (TTP) and disease control rate (DCR). Among investigated SNPs, CYP2W1*6 and CYP2B6*6 correlated with mitotane treatment only in group B. Patients with CYP2W1*6 (*n* = 21) achieved less frequently therapeutic mitotane levels (>14 mg/L) than those with wild type (WT) allele (76.2% vs 51.7%, *p* = 0.051) and experienced shorter TTP (HR = 2.10, *p* = 0.019) and lower DCR (chi-square = 6.948, *p* = 0.008). By contrast, 55% of patients with CYP2B6*6 vs. 28.2% WT (*p* = 0.016) achieved therapeutic range. Combined, a higher rate of patients with CYP2W1*6WT+CYP2B6*6 (60.6%) achieved mitotane therapeutic range (*p* = 0.034). In not completely resectable, recurrent or advanced ACC, CYP2W1*6 SNP was associated with a reduced probability to reach mitotane therapeutic range and lower response rates, whereas CYP2B6*6 correlated with higher mitotane levels. The association of these SNPs may predict individual response to mitotane.

## 1. Introduction

Adrenocortical carcinoma (ACC) is a rare tumor with an estimated incidence between 0.7 and 2.0 per million per year, and characterized by a heterogeneous prognosis mainly depending on initial tumor stage according to the European Network for the Study of Adrenal Tumors (ENSAT) classification [1], resection status [2,3] and Ki67 proliferation index [4,5,6]. Surgery remains the only curative option for localized disease, however, despite complete tumor resection (R0), the rate of local recurrence is high [4,7] and adjuvant treatment with mitotane is frequently recommended [8,9]. Mitotane is approved for unresectable, relapsed or metastatic ACC [10]. It has been recently shown that up to 20% of patients with advanced ACC can experience objective response after mitotane monotherapy, with a progression-free survival (PFS) and an overall survival (OS) of 4.1 and 18.5 months, respectively [11]. Both in adjuvant and palliative treatment, mitotane plasma levels greater than 14 mg/L have been associated with longer survival [11,12,13]. However, mitotane is often associated with several adverse events, including adrenal insufficiency, gastrointestinal-, central nervous system (CNS)- and other endocrine-related effects [8,9].

So far, only few molecular markers have been proposed for predicting the response to mitotane treatment, including ribonucleotide reductase M1 (RRM1) [14] and the cytochrome P450 (CYP) 2W1 [15,16]. Moreover, single nucleotide polymorphism (SNP) of another member of the CYP superfamily, CYP2B6*6, has been proposed to correlate with mitotane plasma levels [17]. However, these molecular markers have been investigated in small cohorts of patients and have not been validated for clinical use.

The CYP superfamily represents the most important enzyme system involved in the biotransformation of endogenous and exogenous substances, including drugs. SNPs in genes coding for members of the CYP superfamily are common and usually affect the enzyme catalytic activity, thereby modifying the drug metabolism, which might result in therapeutic failure or increased adverse reactions [18]. CYP2W1 is one of the most recently described members of the CYP superfamily and it is involved in the metabolism of lysophospholipids, retinoids, aromatic amines and duocarmycin analogues [19]. Interestingly, CYP2W1 is highly expressed in fetal tissues and some tumors, including colon cancer [20], and its expression correlates with more aggressive tumor phenotype and poor outcome [21,22]. We previously showed that ACC patients having high CYP2W1 immunostaining at tumor level present a better response to mitotane therapy in comparison to those with low CYP2W1 staining [15]. These results were very recently confirmed in primary ACC cell culture [16]. Currently, it is unclear whether SNPs of *CYP2W1* might affect the metabolic function of the enzyme [23]. On the other hand, CYP2B6, in addition to CYP34A, represents an important CYP involved in the metabolism of mitotane [24]. The most clinically relevant SNP of this gene is CYP2B6*6 that results in a decreased expression and function of the encoded enzyme [25]. In a previous series of 27 patients with ACC, those harboring the CYP2B6*6 presented higher mitotane plasma levels in comparison to patients with wild type (WT) [17]. However, the correlation between this SNP and the response to mitotane treatment was not investigated.

The aim of this study was to investigate the role of *CYP2W1* and *CYP2B6* SNPs as potential markers of response to mitotane treatment, providing more insights into a personalized mitotane therapy in patients affected by ACC. We also evaluated whether these polymorphisms could have a role in adrenal cancer susceptibility.

## 2. Results

### 2.1. Patient Characteristics and Mitotane Treatment Details

**Figure 1:**
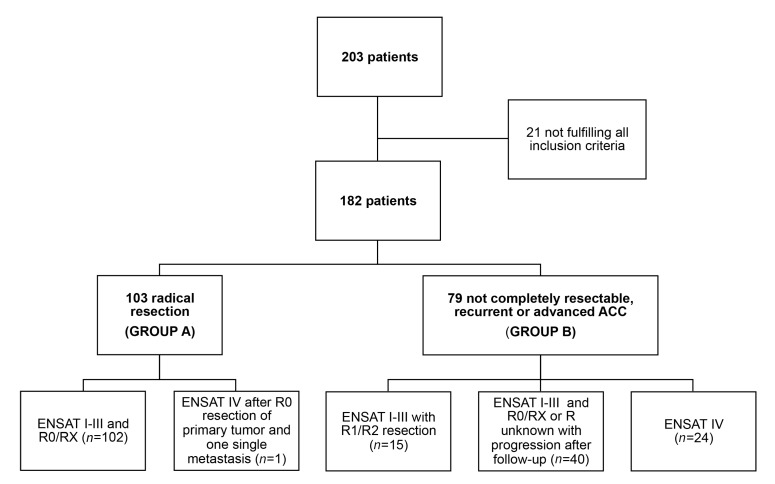
Flowchart of patients with adrenocortical carcinoma included in the present study. Among the entire cohort, 182 patients fulfilled all inclusion criteria and were included in this study. Patients treated with mitotane monotherapy after radical resection were included in group A (*n* = 103). Patients with non-completely resectable, recurrent or advanced ACC and treated with mitotane monotherapy were included in group B (*n* = 79). Abbreviation: R0, complete resection, R1, microscopically not completely resected tumor; R2, macroscopically not completely resected tumor; RX, resection status not assessed.

In total, 182 patients were included in the present study. Group A included 103 patients treated with mitotane as adjuvant therapy after radical tumor resection (R0 and RX) classified at diagnosis as ENSAT stage I-III (*n* = 102) or ENSAT stage IV with a single metastasis (*n* = 1; Figure 1). Group B included 79 patients with either microscopically (R1, *n* = 11) or macroscopically (R2, *n* = 4) residual (after surgery) tumor, or recurrent disease not amenable to radical resection (*n* = 40), or metastatic disease since diagnosis (ENSAT stage IV, *n* = 24; Figure 1).

Clinical and histopathological characteristics at baseline of the included patients (F:M = 121:61; median age at diagnosis 49 years, range 16 to 80 years) are summarized in Table 1. Median follow-up of the entire cohort of patients was 45.7 months (range 5–256). No differences in mean mitotane daily dose were reported between group A and group B (3.2 ± 1.5 vs 3.3 ± 1.7 g/day, respectively, *p =* 0.68). A significantly lower number of patients in group A had disease progression during mitotane treatment compared to those in group B (53, 51.5% vs. 69, 87.3%, chi-square = 26.05, *p <* 0.001).

Patients in group A had a significantly longer median time to progression (TTP) of 34.0 months (95% confident interval (95% CI) 5.13–62.9) under mitotane treatment compared to those in group B, who had a median TTP of 6.0 months (95% CI 3.3–8.7; *p <* 0.001). Similarly, median disease-specific survival (DSS) was significantly longer in group A than group B (median DSS not reached *vs* 38 months (95% CI 15.0–60.9), respectively; *p <* 0.001).

### 2.2. CYP2W1 and CYP2B6 Allele Frequency

The Hardy Weinberg equilibrium (HWE) was calculated within German and Italian subgroups as these were the two major population included in the study (*n* = 117 and 50, respectively) [26]. HWE was not calculated for Greek, Russian, Romanian, Croatian and Serbian patients since the number of included patients originating from these countries was too small (*n* = 6, 4, 3, 1 and 1, for each group). All investigated allele variants were in HWE, except for a slightly excess of homozygotes for CYP2W1*6 in the German population (*p =* 0.03) that it could be due to population stratification [26], and it results not clinically relevant [27].

Regarding CYP2W1*2, 155 patients (85.2%) had WT allele (GG genotype), 26 (14.3%) were heterozygous (GA genotype) and only 1 (0.5%) was homozygous (AA genotype). For CYP2W1*6, 134 cases (73.6%) had WT allele (CC genotype), 40 (22.0%) were heterozygous (CT genotype) and 8 (4.4%) were homozygous (TT genotype). The allele frequencies for CYP2W1*2 and CYP2W1*6 were 8% and 15% respectively, and are comparable to those found in the European population (8% and 17% for CYP2W1*2 and CYP2W1*6, respectively).

Regarding CYP2B6*6, 103 patients (56.7%) had WT allele (GG genotype), 62 (34%) were heterozygous (GT genotype) and 17 (9.3%) were homozygous (TT genotype). For the *CYP2B6* intronic variant, 86 subjects (47.3%) had WT allele (CC genotype), 84 (46.1%) were heterozygous (CT genotype) and 12 (6.6%) were homozygous (TT genotype). The allele frequencies for CYP2B6*6 and *CYP2B6* intronic variant were 26% and 30%, respectively, and were comparable to those found in the European population (28% for both CYP2B6*6 and *CYP2B6* intronic variant).

Due to the low number of homozygous patients, we considered the patients with heterozygosity or homozygosity for each SNPs as one single group (patients with SNP). None of the evaluated SNPs correlated with hormone secretion status, ENSAT tumor stage at diagnosis and ki67 proliferation index.

### 2.3. Achievement of Mitotane Therapeutic Levels

We did not find any correlation between the evaluated SNPs and the achievement of mitotane target levels (≥14 mg/L) in group A (patients treated with mitotane as adjuvant therapy after radical tumor resection).

**Figure 2:**
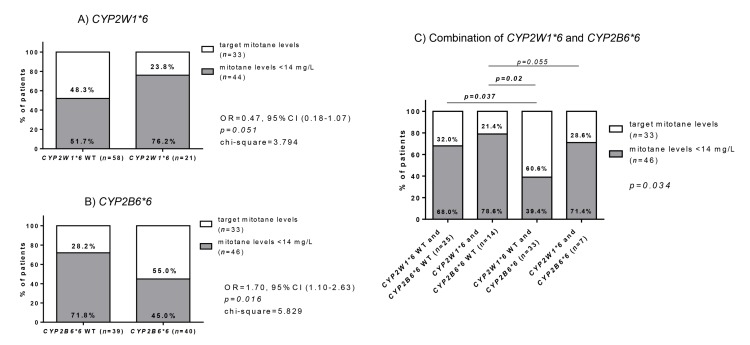
Correlation between the presence of CYP2W1*6 and/or CYP2B*6 SNPs with the achievement of mitotane target levels in group B (*n* = 79). (**A**) Relationship between presence of CYP2W1*6 and achievement of mitotane target levels. (**B**) Relationship between presence of CYP2B6*6 and achievement of mitotane target levels (**C**) Relationship with the achievement of target mitotane levels based on the combination of the two SNPs. Statistical analysis was performed by Chi-square test on absolute values.

In group B, CYP2W1*6 and CYP2B6*6 SNPs correlated with the achievement of mitotane therapeutic levels. We observed that only five patients with CYP2W1*6 (23.8%) achieved mitotane therapeutic range compared to 28 (48.5%) of those with CYP2W1*6 WT (odds ratio (OR) = 0.47, 95% CI 0.18–1.07, chi-square = 3.794, *p = 0.051*; Figure 2A). Opposite to this, the majority of patients with CYP2B6*6 (*n* = 22, 55.0%) achieved mitotane therapeutic levels compared to 11 patients (28.2%) with CYP2B6*6 WT (OR = 1.70, 95% CI 1.10–2.36, chi-square = 5.829, *p = 0.016*; Figure 2B). Accordingly, when we combined both SNPs, a significantly higher proportion of patients with CYP2W1*6 WT and CYP2B6*6 (*n* = 20 out 33, 60.6%) achieved mitotane target levels compared to patients with CYP2W1*6 and CYP2B6*6 WT (*n* = 3 out 14, 21.4%; OR = 5.64, 95% CI 1.32–24.18, *p = 0.02*) and those patients with both SNPs WT (*n* = 8 out 25, 32%; OR = 3.27, 95% CI 1.10–9.75, *p = 0.037*; Figure 2C).

Then we evaluated the potential factor that could predict the achievement of the mitotane therapeutic levels during the TTP by multivariate logistic analyses, including the two SNPs CYP2W1*6 and CYP2B6*6 and the mean mitotane dose during treatment (Table S1). None of the evaluated parameters could predict the achievement of the mitotane target levels in group A, whereas CYP2B6*6 was an independent factor to predict mitotane target levels in group B (OR 2.89, 95% CI 1.06–7.85, *p =* 0.04; Table S1).

### 2.4. Response to Mitotane Treatment

#### 2.4.1. Group A: Patients with Completely Resected Tumor

In line to what observed for the achievement of mitotane target levels, none of the evaluated SNPs correlated with TTP in group A (Table 2). In this group, only the achievement of the mitotane therapeutic range and Ki67 index significantly correlated with TTP at both univariate and multivariate analysis [hazard ratio (HR) = 2.11, 95% CI 1.17–3.82, *p =* 0.013, and HR = 3.25, 95% CI 1.64–6.41, *p =* 0.001, respectively, in the multivariate analysis; Table 2]. Both parameters significantly correlated also with DSS (HR = 2.25, 95% CI 1.02–4.96, *p = 0.046* for the achievement of mitotane target levels; HR = 2.46, 95% CI 1.03–5.87, *p =* 0.043 for Ki67 index; Table 2).

Moreover, we observed that the achievement of the mitotane target levels significantly influence the TTP in patients with CYP2W1*6 or CYP2B6*6 SNPs (Table 3). Particularly, patients with one of the two SNPs and mitotane plasma levels <14 mg/L presented a significantly increased risk of recurrence compared to those who achieved the mitotane target levels (HR = 3.45, 95% CI 1.45–10.42, for patient s with CYP2W1*6 and HR = 3.58, 95% CI 1.22–10.49 for those with CYP2B6*6). A significantly shorter DSS was observed only for patients with CYP2B6*6 (HR = 7.10, 95% CI 1.97–25.60; Table 3).

#### 2.4.2. Group B: Patients with Not Completely Resectable, Recurrent or Advanced ACC

**Figure 3:**
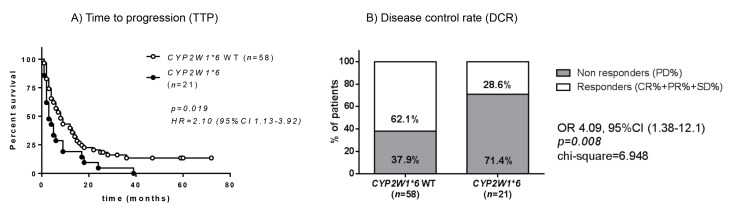
CYP2W1*6 SNP and response to mitotane therapy in group B (*n* = 79). (**A**) Time to progression under therapy (TTP) in patients with CYP2W1*6 compared to those having the wild-type (WT) allele (median TTP 3 vs. 8 months). Statistical analysis by Kaplan-Meier survival curves and Log Rank (Mantel-Cox) test. (**B**) Disease control rate (DCR) according to RECIST 1.1 criteria [28]. DCR was defined as the proportion of patients whose best response was complete response (CR) or partial response (PR) or stable disease (SD) according to RECIST 1.1 criteria. Statistical analysis for DCR was performed by Chi-square test on absolute values.

Conforming to the correlation of CYP2W1*6 SNP with the achievement of mitotane target level in group B, we observed that patients with CYP2W1*6 had a significantly shorter TTP (median TTP 3 months, 95% CI 0.76–5.24) compared to those with CYP2W1*6 WT (median TTP 8 months, 95% CI 5.20–10.80; HR = 2.10, 95% CI 1.13–3.92, *p =* 0.019; Figure 3A). This correlation remained true also after adjusting the survival curve for sex (chi-square 4.45, *p =* 0.035), age (chi-square = 5.21, *p =* 0.02) and ENSAT stage at diagnosis (chi-square = 4.67, *p =* 0.031).

However, at multivariate analysis, including the achievement of the mitotane target levels and the time to progression pre-mitotane treatment (preM-TTP), considered as the time from ACC diagnosis to first radiological evidence of recurrence or progression, the CYP2W1*6 SNP was not more significant (HR = 1.54, 95% CI 0.91–2.61, *p =* 0.10; Table 4).

Evaluating the best overall response to mitotane according to RECIST criteria [28], we observed that among patients with CYP2W1*6 SNP, only 1 patient (4.8%, 95% CI 0.1–23.8) achieved complete response (CR) and 1 achieved partial response (PR) after treatment compared to 4 patients (6.9%, 95% CI 1.9–16.7) and 7 patients (12.1%, 95% CI 5.0–23.3), respectively, with CYP2W1*6 WT (Table 5). Disease control rate (DCR) was 71.4% in CYP2W1*6 SNP compared to 37.9% of those with CYP2W1*6 WT (OR = 4.09, 95% CI 1.38–12.1, chi-square = 6.948, *p* = 0.008; Figure 3B).

On the other side, CYP2B6*6 SNP showed no association with TTP. In fact, no difference was observed between patients with CYP2B6*6 (median TTP 8 months, 95% CI 4.92–11.08) and those with CYP2B6*6 WT (median TTP 5 months, 95% CI 2.38–7.62; HR = 0.85, 95% CI 0.52–1.39, *p =* 0.52). Similar to this, CYP2B6*6 SNP did not correlate with DCR (OR = 1.31, 95% CI 0.54–3.19, chi-square = 0.36, *p =* 0.55).

The combination of CYP2W1*6 and CYP2B*6 significantly correlated with TTP (chi-square = 4.27, *p =* 0.039; Figure S1). Particularly, patients with CYP2W1*6 and CYP2B6*6 WT (*n* = 14) had a significantly shorter TTP (median TTP 3 months, 95% CI 1.79–4.21) compared to those with CYP2W1*6 WT and CYP2B6*6 WT (*n* = 25, median TTP 9 months (95% CI 0–18.79), HR = 3.0 (95% CI 1.51–5.94), *p =* 0.035) and to those with CYP2W1*6 WT and CYP2B6*6 (*n* = 33, median TTP 8 months (95% CI 5.20–10.80)*,* HR = 2.67 (95% CI 1.40–5.06), *p =* 0.014). No difference was found compared to patients with CYP2W1*6 and CYP2B6*6 (*n* = 7, median TTP 4 months (95% CI 0–9.13), HR = 1.33 (95% CI 0.54–3.30), *p =* 0.38).

Interestingly, as observed for group A, the achievement of the mitotane target levels significantly influenced the TTP of patients with CYP2W1*6 or CYP2B6*6 SNPs (Table 6). In particular, patients with CYP2W1*6 or CYP2B6*6 SNPs who not achieved the mitotane range presented a significantly increased risk of progression compared to those who had mitotane levels within the target range (HR = 3.52, 95% CI 1.16–10.65 and HR = 5.59, 95% CI 2.40–13.00). A significantly shorter DSS was observed only for patients with CYP2B6*6 who did not achieve the therapeutic target mitotane levels (HR = 3.89, 95% CI 1.61–9.43; Table 6).

In this group of patients with advanced disease, none of the evaluated SNPs correlated with the DSS (Table 4). Only Ki67 index correlated with DSS at both univariate and multivariate analysis (HR = 2.07, 95% CI 1.05–4.10, *p =* 0.037 at multivariate analysis; Table 4).

### 2.5. Adverse Events Associated to Mitotane Treatment

Mitotane-related adverse events were similar in the two groups (chi-square = 17.88, *p* = 0.53), and the most frequent adverse event was an increase of GGT reported in 94.5% and 93% of patients in group A and B, respectively. None of the evaluated SNPs correlated with the type of adverse events (chi-square = 8.78 *p* = 0.98 for CYP2W1*2; chi-square = 23.55, *p* = 0.21 for CYP2W1*6; chi-square = 23.15, *p* = 0.23 for CYP2B6*6; chi-square = 17.61, *p =* 0.0.55 for *CYP2B6* intronic variant) or with the number of adverse events (Eta-square coefficient was: 0.005 for CYP2W1*2; 0.006 for CYP2W1*6; 0.00009 for CYP2B6*6; 0.0005 for *CYP2B6* intronic variant).

### 2.6. Mitotane Metabolites and Correlation with CYP2W1*6 and CYP2B6*6 SNPs

In a subgroup of 29 patients with available plasma samples (14 in group A and 15 in group B), we measured mitotane metabolites 1,1-(*o*,*p*′-dichlorodiphenyl)-2,2 dichloroethene (*o*,*p*′-DDE) and 1,1-(*o*,*p*′-dichlorodiphenyl) acetic acid (*o*,*p*′-DDA) together with mitotane (*o*,*p*′-DDD), and correlated them with CYP2W1*6 and CYP2B6*6 SNPs.

In group A, we observed that patients with CYP2B6*6 (*n* = 5) had significant higher levels of *o*,*p*′-DDE (median levels 3.45 µg/mL (range 1.54–4.37)) compared to patients with CYP2B6*6 WT (*n* = 9; median levels 0.88 µg/mL (range 0.02–3.57), fold change 2.58 (95% CI −0.05–3.44), *p =* 0.04; Supplementary Figure S2A). No correlations were found between CYP2B6*6 SNP and *o*,*p*′-DDA or *o*,*p*′-DDD (Figure S2B,C). However, no correlations were shown between CYP2W1*6 SNP and mitotane metabolites.

In group B, patients with CYP2W1*6 (*n* = 5) had significant lower levels of *o*,*p*′-DDE [median levels 0.17 µg/mL (range 0–1.61)] compared to patients with CYP2B6*6 WT [*n* = 10; median levels 1.92 µg/mL (range 0.06–7.05), fold change −1.74 (95% CI −4.04 to 0.11), *p =* 0.039; Supplementary Figure S2D). A trend to lower *o*,*p*′-DDA levels were also observed in patients with CYP2W1*6 (median levels 3.50 µg/mL (range 1.20–10.39) vs 16.74 µg/mL (range 3.25–44.96), fold change −13.25 (95% CI −25.49 to 0.50), *p =* 0.058; Figure S2E). No significant correlation was observed for *o*,*p*′-DDD levels and CYP2W1*6 (Figure S2F), as well as between CYP2B6*6 SNP and mitotane metabolites.

## 3. Discussion

ACC is a rare tumor with heterogeneous prognosis [1,9] and the only approved drug by FDA and EMA for the treatment of advanced disease is mitotane [10,11]. It has been demonstrated that mitotane levels of 14 mg/L or greater are significantly associated with longer survival [11,12,13]. This was confirmed also in our study, where the achievement of the mitotane target levels represented an independent and, together with Ki67 index, the strongest predictor of tumor recurrence or progression both in patients after radical resection and in those with not completely resected, recurrent or advanced ACC. For that reason, the achievement of the target range should be considered as the goal of the mitotane treatment [9]. Currently, no one of the proposed biomarkers, which were previously described to predict response and attainment of therapeutic plasma concentrations [14,15,16,17], is used in clinical practice since they have not been validated. In this multicenter study, we evaluated SNPs of *CYP2W1* and *CYP2B6* as potential predictive markers of response to mitotane in a large cohort of patients treated with mitotane monotherapy as first pharmacological therapy either after radical resection (group A) or in not completely resectable, recurrent or advanced ACC (group B).

First, we showed that all investigated SNPs were in Hardy-Weinberg Equilibrium in our cohort of ACC, except for a slightly deviations toward an excess of homozygotes for the CYP2W1*6 in German patients. This slight discrepancy is not clinically relevant [27] and could be due to population stratification [26], such as assortative mating. Thus, we can confirm that there is no association between the investigated SNPs and an increased ACC risk, as already reported for colon cancer [23].

Among the investigated SNPs, only CYP2W1*6 and CYP2B6*6 SNPs revealed to be associated with mitotane plasma levels. These results are in accordance to previous studies that reported an association between the investigated SNPs and an increase or decrease metabolism of different drug [23,29]. Moreover, we validated the association between CYP2B6*6 and higher mitotane plasma levels in a larger cohort of ACC patients [17]. We demonstrated that both SNPs were significantly associated with achievement of mitotane target levels. Particularly, the majority of patients with CYP2W1*6 did not achieved the mitotane therapeutic range, whereas most patients with CYP2B6*6 attained the target levels, showing an opposite effect. This was evident also when we combined the two SNPs. Moreover, we observed that patients with CYP2W1*6 had a significantly shorter TTP and a poorer response to mitotane therapy compared to those with WT allele. However, this association was not significant in a multivariate analysis, suggesting that CYP2W1*6 has more likely an indirect effect on the response to mitotane treatment through influencing the achievement of mitotane target levels. In this study, the role of CYP2W1*6 and CYP2B6*6 on mitotane treatment was evident only in patients with not completely resectable, recurrent or advanced ACC (group B). The discrepancy observed between two groups could be due to the presence of the tumor itself. In fact, we already demonstrated that CYP2W1 is highly expressed in ACC [15] at both RNA and protein levels, and it could be locally (at tumor level) involved in mitotane metabolism, thus impacting the achievement of the target range. In patients after radical resection (group A), there is no local metabolism of mitotane, explaining the lack of effect of *CYP2W1* phenotype on mitotane levels. We assumed that a similar mechanism could be the case for the *CYP2B6* phenotypes. While no one demonstrated until now that this cytochrome is overexpressed in ACC, there is however evidence of gene amplifications associated with ACC [30].

To better investigate the potential role of the CYP2W1*6 and CYP2B6*6 SNPs on mitotane metabolism, we measured two mitotane metabolites. We found that *o*,*p*′-DDE levels positively correlated with CYP2B6*6 and negatively with CYP2W1*6. Although the antitumor activity of mitotane metabolites is controversial [12,31,32,33], *o*,*p*′-DDE, together with *o*,*p*′-DDA, reflects the metabolism of mitotane. Thus, an alteration of o-p’-DDE plasma levels might mirror an alteration of mitotane metabolism. Therefore, a decrease of enzymatic activity associated with CYP2B6*6 SNPs might result in an increase of mitotane plasma levels as already demonstrated for other drugs, such as efavirenz [34]. On the other hand, the effect of *CYP2W1* SNPs is currently unclear and, contrary to what has been described for duocarmycin analogs in colon cancer cells [23], CYP2W1*6 may have a different catalytic capacity for mitotane metabolism.

With this study we confirmed that the achievement of the mitotane therapeutic range is the most remarkable factor to predict the response to mitotane treatment both in adjuvant setting and in recurrent/advanced ACC [12], as well as to predict the DSS after radical tumor resection [11,12]. We were also able to confirm the relevant role of clinical and histopathological parameters, including ENSAT tumor stage, preM-TTP, Ki67 index, R status, and the hormonal status as significant prognostic markers of survival [1,2,4,5,6,35].

Due the rarity of ACC, it appears difficult to perform prospective studies including a large number of patients homogeneously treated, and till now only retrospective studies were performed [8,11,13,14,15,17,36]. The only prospective study evaluating the efficacy of mitotane in adjuvant treated patients has just stop recruitment (ClinicalTrials.gov Identifier: NCT00777244). In our study, thanks to its multicenter design, we were able to include a very large number of patients treated with mitotane monotherapy as first-line medical treatment (*n* = 182). To the best of our knowledge, this is the largest described cohort of patients with these characteristics and this represents a major strength of the study. Due to the retrospective nature of the study, we were not able to evaluate the difference between the high-starting dose and low-starting dose mitotane regimen, which is certainly a limitation. However, the fact that we included patients treated with both standard regimens reflects the reality in the expert centers around the globe, because none of them has been proven to be superior. The high-dose starting regimen led to only a slightly but not significantly higher mitotane plasma levels within the first 12 weeks of treatment [36] and in the clinical practice, mitotane dosage is adjusted according to blood concentrations and patient tolerability after the first 2–4 weeks of therapy [9,13]. Another important point of this study is that the genotyping of CYP2W1*6 and CYP2B6*6 SNPs is a cost-effective analysis that may be done by the majority of labs and thus is easily applicable in the clinical practice. Moreover, the genotyping of SNPs could be performed before starting mitotane, allowing clinicians to pre-select those patients with advanced ACC who may not respond to mitotane and who may for instance better benefit from more aggressive treatment (i.e., standard chemotherapy). This kind of patients’ pre-selection is currently not possible with any other proposed predictive biomarkers of response and could lead in the near future to an innovative personalized medicine approach in the treatment of ACC patients. However, our findings should be further validated in a prospective study. Moreover, functional *in vitro* experiments should be performed to better investigate the role of CYP2W1*6 and CYP2B6*6 on mitotane metabolism.

## 4. Materials and Methods

### 4.1. Study Design and Population

We performed a multicenter retrospective study including ACC patients from 6 ENSAT centers (www.ensat.org): two centers from Germany, three from Italy and one from Greece. We included only Caucasian patients treated with mitotane monotherapy as first line medical treatment after radical resection or in advanced disease. Other inclusion criteria were: availability of clinical and histopathological data at diagnosis and during follow-up, and availability of whole blood sample for genotyping. Exclusion criteria included previous or concomitant treatment with systemic cytotoxic therapies, age at diagnosis less than 16 years old, incomplete follow-up and/or incomplete details of mitotane treatment. Samples have been stored in the Interdisciplinary bank of biomaterial and data Würzburg (IBDW) [37]. In total, we collected samples and data of 203 (F/M = 133/70) ACC patients who had been treated with mitotane monotherapy between January 2002 and December 2017 (last follow-up). However, 21 patients did not fulfill all inclusion criteria and were excluded from further analysis.

The study was approved by the local Ethics Committees of the University of Wuerzburg, Germany (approval number 88/11) and was conducted in accordance with the principles of the Declaration of Helsinki. Written informed consent was obtained from all patients.

### 4.2. Clinical Data, Mitotane Treatment and Endpoints Assessment

The evaluated clinical and histopathological characteristics of the patients at baseline included sex, age at diagnosis, country of origin, tumor size initial tumor stage according to ENSAT classification [1], hormonal secretion pattern, Weiss score, Ki67 proliferation index and R status. All patients received the same mitotane formulation (Lysodren^®^, 500 mg tablets, from HRA Pharma, Paris, France), starting with either a low-dose or a high-dose protocol according to standard clinical practice in each center [9,36]. Treatment was discontinued upon discretion of the local physician/investigator in case of unacceptable toxicity, evidence of disease progression, or patients’ decision. During follow-up, all patients underwent standardized follow-up every 3–6 months according to disease status [9]. These evaluations included physical examination, biochemical workup and imaging procedures, including computed tomography (CT) of chest and abdomen or fluorodeoxyglucose (FDG)-positron emission tomography (PET)/CT. The baseline characteristics as well as data on mitotane treatment and follow-up were collected through the ENSAT Registry (www.ens@t.org/registry).

We divided the patients in two groups according to tumor resection, ENSAT stage and disease status: group A and group B. In group A we included patients who underwent radical resection of the primary tumor and, in case of metastatic disease, together with complete metastasis resection and who were treated adjuvantely with mitotane. In group B we included patients with not completely resectable, recurrent or advanced ACC who were treated with mitotane as first medical therapy. All endpoints were evaluated separately for in group A and group B. The major endpoint was the correlation between the presence of investigated SNPs and the achievement of the mitotane therapeutic levels, defined by mitotane levels ≥14 mg/L for at least half of the treatment period. Mitotane levels were routinely monitored in patients during the follow-up through the Lysosafe service (www.lysosafe.com). Secondary endpoint included the response to mitotane treatment evaluated by TTP and DSS under therapy. TTP was defined as the time from the start of mitotane until the first radiological evidence of disease relapse or progression, or last follow-up or treatment discontinuation. DSS was defined as the time from mitotane treatment start to disease-related death or last follow-up. In patients with not completely resectable, recurrent or advanced disease we evaluated also the DCR, defined as the proportion of patients whose best response was CR or PR or stable disease (SD) according to RECIST 1.1 criteria [28].

### 4.3. Genotyping and Sequencing of CYP2W1 and CYP2B6 Polymorphisms

We genotyped two *CYP2W1* SNPs and two *CYP2B6* SNPs, according to data available from literature [17,23]. Specifically, we investigated for *CYP2W1* (ENSG00000073067: ENST00000308919, transcript position in coding sequence (CDS)): CYP2W1*2 (rs3735684, c.541G > A, p.A181T) and CYP2W1*6 (rs3808348, c.1463C > T, p.P488L). The polymorphisms investigated for *CYP2B6* (ENSG00000197408: ENST00000643956, position in CDS) were: CYP2B6*6 (rs3745274, c.516G > T, p.Q172H) and *CYP2B6* intronic variant (rs4803419, c.485-18C > T).

DNA was extracted from 2 mL of whole blood previously collected in ETDA tubes and stored at −80 °C, using NucleoSpin^®^ Blood L (#740954.20, Macherey-Nagel, Düren, Germany) following the manufacturer’s instructions. The investigated polymorphisms were genotyped by Polymerase Chain Reaction (PCR) using oligonucleotide primers of approximately 18–20 base pairs (Table S2). 30 ng of genomic DNA was used for PCR amplification, which was performed in a final volume of 25 μL containing 1.5 mM MgCl_2_, 0.2 µM of each primer, 200 μM dNTPs and 1 U Taq DNA Polymerase. Cycling conditions were: 95 °C for 5 min followed by 35 cycles of denaturing at 94 °C (20 s), annealing at 58 or 60 °C (30 s) depending on primers, and elongation at 72 °C (1 min). Direct sequencing of PCR products was performed according to Sanger [38] using the QuickStart Cycle Sequencing Kit (#608120, AB Sciex, Beckman Coulter, Krefeld, Germany) on a CEQ8000 DNA Analyzer (AB Sciex).

### 4.4. Evaluation of Mitotane Related Adverse Events

Potential mitotane therapy related adverse events were regularly registered during the follow up visits and assessed according the Common Terminology Criteria for Adverse Events (CTCAE) version 4.03 [39]. According to the known spectrum of adverse effects of mitotane [8,9], we searched the medical records for gastrointestinal (diarrhea, nausea, vomiting, flatulence, abdominal pain), CNS-related (concentration impairment, confusion, amnesia, aphasia, depression, ataxia, dizziness, vertigo, dysarthria, dysgraphia, dysgeusia), hepatic (increase of γ-glutamyl transferase, and aspartate or alanine aminotransferase), endocrine (adrenal insufficiency, hypothyroidism, hypogonadism), metabolic (increase of cholesterol levels), hematologic (leukopenia), dermatologic (rash, dry skin, sweating), and musculoskeletal adverse events (myalgia, muscle cramps or pain), as well as constitutional (asthenia, fatigue), ocular (visus alterations, blurred vision, conjunctivitis, retinal vascular disorders), and other unspecific symptoms (dyspnea, fever, insomnia, restlessness infections, sepsis). The occurrence of the different mitotane related adverse events, as well as the total numbers of observed events, were then associated with all investigated SNPs.

### 4.5. Measurement of Mitotane Metabolites

For a subgroup of 29 patients we had available additional plasma sample collected during mitotane treatment and stored at −80 °C for the measurement of *o*,*p*′-DDD and its two principal plasma metabolites, *o*,*p*′-DDE and *o*,*p*′-DDA. This analysis was carried out by a validated high-performance liquid chromatography method coupled with ultraviolet detection (HPLC-UV), as previously described [40].

### 4.6. Statistical Analysis

The allele frequencies were compared to those found in the European population using the 1000 Genomes Project Phase 3 allele frequencies (ENSEMBLE, http://www.1000genomes.org/category/ frequently-asked-questions/population) and the HWE equilibrium was calculated within each country of origin [26].

The Fisher’s exact test or the Chi-square test were used to investigate dichotomic variables, while a two-sided t test (or non-parametric test) was used to test continuous variables as appropriate. A non-parametric Kruskal-Wallis test, followed by Bonferroni post-hoc test, was used for comparison among several groups for non-normal distributed variables. Correlations and 95% CI between different parameters were evaluated by linear regression analysis. Association between dichotomic and continuous variables were evaluated by Eta correlation. All survival curves were obtained by Kaplan-Meier estimates and the differences between survival curves were assessed by the Log Rank (Mantel-Cox) test. Univariate analysis followed by multivariate regression analysis were performed by Cox proportional hazard regression model to identify those factors that might independently influence survival. In survival and regression analyses we considered all the investigated SNPs as well as clinical-pathological parameters that have been describe to have a prognostic role in ACC patients, including the achievement of mitotane therapeutic range (as previously defined) [12], Ki67 index (cut-off ≥ 20%) [4,41], hormonal secretion (cortisol secreting alone or in combination with other steroids *vs* other steroids-secreting or inactive) [42], and age (cut-off ≥ 50 years) [5,6]. The ENSAT tumor stage (I-II *vs* III-IV in patients after radical resection of primary tumor and, in one case, after radical resection of primary tumor and associated single liver metastasis) [1] was evaluated in the group treated adiuvantly with mitotane. In patients with not completely resectable, recurrent or advanced disease, we considered preM-TTP, evaluated as the time from ACC diagnosis to first radiological evidence recurrence or metastasis. According to preM-TTP, patients were divided in three categories: preM-TTP ≤ 3 months, between 3 and 24 months, and ≥24 months. The tumor resection status (complete resection (R0) or not assessed (RX) *vs* microscopic (R1) or macroscopic (R2) incomplete resection) [2] was evaluated only in the group of patients with advanced disease, since, by definition, in the group of patients who had radical resection did not include patients with R1 or R2 resection. To evaluate the predictor factors of mitotane plasma concentration we performed a multivariate logistic regression analysis and OR were calculated with 95% CI. Statistical analyses were made using GraphPad Prism (version 5.0, La Jolla, CA, USA) and SPSS Software (PASW Version 21.0, SPSS Inc., Chicago, IL, USA). *p* values *<* 0.05 were considered as statistically significant.

## 5. Conclusions

We suggest that the association of CYP2W1*6 and CYP2B6*6 may be used to predict the individual response to mitotane treatment. Our study provides the basis for a clinical trial to test the value of genotyping for CYP2W1*6 and CYP2B6*6 SNPs in ACC patients treated with mitotane monotherapy.

## Figures and Tables

**Table 1:** Baseline clinical and histopathological characteristics of the entire cohort of patients with adrenocortical carcinoma and in group A and B.

Baseline Characteristics of the Patients					
Parameter	All Patients	Group A	Group B	*p*	Chi-Square
*n*	182	103	79	-	-
Sex					
F	121	68 (66%)	53 (67.1%)	0.88	0.023
M	61	35 (34%)	26 (32.9%)		
Age yrs	49 (16–80)	47 (18–75)	51 (16–80)	**0.027**	-
Tumor size cm	10 (2–24)	10 (2–24)	11 (3–24)	0.05	-
Hormone secretion				**0.007**	16.02
Cortisol (alone or with other steroids)	80 (44.0%)	37 (35.9%)	43 (54.4%)		
Androgens	15 (8.2%)	14 (13.6%)	1 (1.3%)		
Aldosterone	4 (2.2%)	4 (3.9%)	0 (0%)		
Estrogens	2 (1.1%)	1 (1.0%)	1 (1.3%)		
Inactive	53 (29.1%)	33 (32.0%)	20 (25.3%)		
Unknown	28 (15.4%)	14 (13.6%)	14 (17.7%)		
ENSAT Tumor Stage at diagnosis				**<0.001**	35.45
I–II	108 (59.3%)	75 (72.8%)	33 (41.8%)		
III	49 (27.0%)	27 (26.2%)	22 (27.8%)		
IV	25 (13.7%)	1 (1.0%)	24 (30.4%)		
Resection Status				**<0.001**	56.60
R0	114 (62.6%)	83 (80.6%)	31 (39.2%)		
RX	34 (18.7%)	20 (19.4%)	14 (17.7%)		
R1/R2	28 (15.4%)	0 (0%)	28 (15.4%)		
unknown	6 (3.3%)	0 (0%)	6 (7.6%)		
Ki67% proliferation index ^a^	17 (1–90)	20 (1–90)	10 (1–60)	0.076	-
Weiss score ^b^	6 (2–9)	6 (2–9)	6 (3–9)	0.19	-

Data were reported as total number and percentage of patients or as median value and range. ^a^ Ki67%: for 12 patients Ki67% was unknown (6 for each group). ^b^ Weiss score: only one patient had a Weiss score = 2, but it was associated with a high proliferation index (Ki67% = 20%). Statistical analyses between group A and group B were performed by Mann-Whitney test and Chi-square test for continuous or dichotomic variables, respectively. A *p* value in bold type denotes a significant difference (*p* < 0.05). Legend: F, female; M, male; n, number of patients; Resection Status: 0, complete resection; 1, microscopically not completely resected tumor; 2, macroscopically not completely resected tumor; X, not assessed; yrs, years.

**Table 2:** Univariate and multivariate analysis correlating clinical and histopathological parameters and the investigated SNPs with response to mitotane treatment and disease-specific survival in group A (*n* = 103).

Group A								
Parameters	TTP				DSS			
	Univariate		Multivariate		Univariate		Multivariate	
	*p*	HR (95% CI)	*p*	HR (95% CI)	*p*	HR (95% CI)	*p*	HR (95% CI)
Mitotane therapeutic range	**0.024**	1.92 (1.09–3.37)	**0.019**	2.04 (1.13–3.71)	**0.032**	2.38 (1.08–5.26)	**0.046**	2.25 (1.02–4.96)
ENSAT stage at diagnosis	0.13	1.57 (0.88–2.79)	n.a	-	0.77	1.44 (0.48–2.96)	n.a	-
Ki67 index	**<0.001**	3.46 (1.76–6.78)	**0.001**	3.06 (1.55–6.05)	**0.026**	2.68 (1.13–6.35)	**0.043**	2.46 (1.03–5.87)
Hormonal secretion	0.055	1.8 (0.99–3.30)	n.a.	-	0.08	2.22 (0.92–5.39)	n.a.	-
Age at diagnosis	0.93	1.03 (0.59–1.78)	n.a.	-	0.51	0.79 (0.21–1.31)	n.a.	-
CYP2W1*2	0.15	0.54 (0.23–1.26)	n.a.	-	0.12	0.21 (0.03–1.53)	n.a.	-
CYP2W1*6	0.39	1.30 (0.71–2.34)	n.a.	-	0.79	1.12 (0.49–2.56)	n.a.	-
CYP2B6*6	0.77	0.92 (0.52–1.61)	n.a.	-	0.63	1.21 (0.56–2.58)	n.a.	-
*CYP2B6* intronic variant	0.07	0.61 (0.35–1.05)	n.a.	-	0.29	0.66 (0.31–1.42)	n.a.	-

Group A includes patients who were treated with mitotane as adjuvant therapy after radical tumor resection. For ENSAT tumor stage, stage I–II *vs.* III–IV (local advanced/metastasized) was considered. For Ki67 index, a cut-off of 20% was considered. For hormonal secretion, cortisol alone or in combination vs other steroids or inactive tumor was considered. Statistical analyses were performed by Cox proportional hazard regression and for the multivariate models were considered only those parameters that were statistically significant at the univariate analysis. A *p* value in bold type denotes a significant difference (*p <* 0.05). Abbreviations: 95% CI, 95% confidence intervals, DSS, disease specific survival; HR, hazard ratio; n.a., not applicable; TTP, time to progression under therapy.

**Table 3:** Time to progression and disease-specific survival for patients with CYP2W1*6 or CYP2B6*6 correlating with the achievement of the mitotane target levels in group A.

Group A (*n* = 98) *							
SNPs	*n*	TTP			DSS		
		Median TTP Months	HR (95% CI)	*p*	Median DSS Months	HR (95% CI)	*p*
CYP2W1*6							
target levels	15	Not reached			Not reached		
mitotane levels <14	12	10.5	3.45 (1.45–10.42)	**0.03**	60.0	2.72 (0.65–11.3)	0.17
CYP2W1*6 WT							
target levels	36	66.0			Not reached		
mitotane levels <14	35	22.0	1.61 (0.81–0.65)	0.18	Not reached	2.40 (0.93–6.21)	0.07
CYP2B6*6							
target levels	23	66.0			Not reached		
mitotane levels <14	14	5.0	3.58 (1.22–10.49)	**0.02**	60.0	7.10 (1.97–25.60)	**0.003**
CYP2B6*6 WT							
target levels	28	53.0			Not reached		
mitotane levels <14	33	19.0	1.57 (0.77–3.20)	0.22	Not reached	1.30 (0.47–3.61)	0.32

Target mitotane levels was defined when the mitotane blood level was ≥14 mg/L for at least half of the treatment period. Statistical analysis was performed by Kaplan-Meier and the differences between the groups by Log Rank (Mantel-Cox) test. A *p* value in bold type denotes a significant difference (*p <* 0.05). Abbreviations: 95% CI, 95% confidence intervals, DSS, disease specific survival; HR, hazard ratio; TTP, time to progression under therapy. * 5 patients among the entire group A (*n* = 103) had missing data and were not included in this analysis.

**Table 4:** Univariate and multivariate analysis correlating clinical and histopathological parameters and the investigated SNPs with the response to mitotane treatment and the disease-specific survival in group B (*n* = 79).

Group B								
Parameters	TTP				DSS			
	Univariate		Multivariate		Univariate		Multivariate	
	*p*	HR (95% CI)	*p*	HR (95% CI)	*p*	HR (95% CI)	*p*	HR (95% CI)
Mitotane therapeutic range	**0.003**	2.11 (1.84–3.47)	**0.002**	2.29 (1.37–3.85)	0.77	1.69 (0.94–3.04)	n.a.	-
PreM-TTP	**0.012**	0.67 (0.49–0.92)	**0.004**	0.64 (0.47–0.87)	**0.004**	1.65 (1.17–2.32)	0.13	0.68 (0.42–1.12)
Ki67 index	0.057	1.63 (0.99–2.69)	n.a.	-	**<0.001**	3.07 (1.67–5.65)	**0.037**	2.07 (1.05–4.10)
R status	0.24	1.35 (0.81–2.25)	n.a.	-	**<0.001**	3.34 (1.80–6.20)	0.09	1.92 (0.88–4.16)
Hormonal secretion	0.21	0.70 (0.40–1.22)	n.a.	-	0.19	0.65 (0.35–1.23)	n.a.	-
Age at diagnosis	0.97	0.99 (0.61–1.59)	n.a.	-	0.78	0.93 (0.53–1.61)	n.a.	-
CYP2W1*2	0.97	1.01 (0.52–1.98)	n.a.	-	0.65	1.18 (0.57–2.44)	n.a.	-
CYP2W1*6	**0.028**	1.78 (1.06–2.99)	*0.10*	1.54 (0.91–2.61)	0.36	1.32 (0.72–2.43)	n.a.	-
CYP2B6*6	0.54	1.01 (0.52–1.98)	n.a.	-	0.80	1.08 (0.62–1.88)	n.a.	-
*CYP2B6* intronic variant	0.49	1.18 (0.73–190)	n.a.	-	0.88	1.04 (0.59–1.83)	n.a.	-

Group B includes patients with not completely resected tumor, or recurrent disease not amenable to radical resection or metastatic disease at diagnosis who were treated with mitotane monotherapy. For the time from ACC diagnosis to recurrence or metastasis, time to progression pre-mitotane treatment (preM-TTP) ≤3 months, between 3 and 24 months, and ≥24 month were considered. For Ki67 index, a cut-off of 20% was considered. For R status at diagnosis, R0/RX vs. R1/R2 was considered. For hormonal secretion, cortisol alone or in combination vs other steroids or inactive tumor was considered. Statistical analyses were performed by Cox proportional hazard regression and for the multivariate models were considered only those parameters that were statistically significant at the univariate analysis. A p value in bold type denotes a significant difference (*p* < 0.05). Abbreviations: 95% CI, 95% confidence intervals, DSS, disease specific survival; HR, hazard ratio; n.a., not applicable; preM-TTP, time to progression pre-mitotane treatment; R, resection status; TTP, time to progression under therapy.

**Table 5:** Best overall response to mitotane treatment according to RECIST 1.1 criteria in group B (*n* = 79).

Best Response	CYP2W1*6 WT (*n* = 58)		CYP2W1*6 (*n* = 21)	
	*n*	% (95% CI)	n	% (95% CI)
Complete response	4	6.9% (1.9–16.7)	1	4.8% (0.1–23.8)
Partial response	7	12.1% (5.0–23.3)	1	4.8% (0.1–23.8)
Stable disease	25	43.1% (30.2–56.8)	4	19.0% (5.4–41.9)
Progressive disease	22	37.9% (25.5–51.6)	15	71.4% (47.8–51.6)

Patients with not completely resected tumor, or recurrent disease not amenable to radical resection or metastatic disease at diagnosis (group B) were divided according the CYP2W1*6 SNP. The objective tumor response for target lesions was evaluated according to RECIST 1.1 criteria [28] as follow: complete response as the disappearance of all target lesions; partial response when at least a 30% decrease in the sum of diameters of target lesions; progressive disease when at least a 20% increase in the sum of diameter of target lesions; stable disease when neither sufficient shrinkage to qualify for partial response nor sufficient increase to qualify for progression. Abbreviations: 95% CI, 95% confidence intervals, n, number of patients; SNP, single nucleotide polymorphism.

**Table 6:** Time to progression and disease-specific survival for patients with CYP2W1*6 or CYP2B6*6 correlating with the achievement of the mitotane target levels in group B.

Group B (*n* = 79)							
SNPs	*n*	TTP			DSS		
		Median TTP Months	HR (95% CI)	*p*	Median DSS Months	HR (95% CI)	*p*
CYP2W1*6							
target levels	5	18.0			115.0		
mitotane levels <14	16	3.0	3.52 (1.16–10.65)	**0.03**	36.0	1.67 (0.52–5.39)	0.39
CYP2W1*6 WT							
target levels	28	13.0			75		
mitotane levels <14	30	5.0	1.95 (1.07–3.55)	**0.03**	40	1.45 (0.73–2.86)	0.23
CYP2B6*6							
target levels	22	15.0			75.0		
mitotane levels <14	18	4.0	5.59 (2.40–13.00)	**<0.0001**	25.0	3.89 (1.61–9.43)	**0.003**
CYP2B6*6 WT							
target levels	11	13.0			50.5		
mitotane levels <14	28	4.5	1.37 (0.34–2.91)	0.42	58.0	0.99 (0.39–2.43)	0.95

Target levels for mitotane was defined when the mitotane blood level was ≥14 mg/L for at least half of the treatment period. Statistical analysis was performed by Kaplan-Meier and the differences between the groups by Log Rank (Mantel-Cox) test. A *p* value in bold type denotes a significant difference (*p* < 0.05). Abbreviations: 95% CI, 95% confidence intervals, DSS, disease specific survival; HR, hazard ratio; TTP, time to progression under therapy.

## Supplementary Materials

The following are available online at https://www.mdpi.com/2072-6694/12/2/359/s1, Figure S1: Combination of CYP2W1*6 and CYP2B6*6 SNP and response to mitotane therapy in group B (*n* = 79), Figure S2: Correlation between mitotane metabolites measured by HPLC-UV and presence of *CYP2W1*6* and *CYP2B6*6* SNPs, Table S1: Multivariate logistic regression analyses predicting plasma mitotane therapeutic levels, Table S2: PCR Primers sequences used for detecting *CYP2W1* and *CYP2B6* investigated single nucleotide polymorphisms.
