# Supplementary material for: Effects of Germline CYP2W1*6 and CYP2B6*6 Single Nucleotide Polymorphisms on Mitotane Treatment in Adrenocortical Carcinoma: A Multicenter ENSAT Study
Source: Cancers (Basel). 2020 Feb 4;12(2):359. doi: 10.3390/cancers12020359 (PMC7072643; doi:10.3390/cancers12020359)
Supplement: Supplementary file 1 [file cancers-12-00359-s001.pdf]

Supplementary

Effects of Germline CYP2W1*6 and CYP2B6*6 Single Nucleotide Polymorphisms on Mitotane Treatment in Adrenocortical Carcinoma: A Multicenter ENSAT Study

Barbara Altieri, Silviu Sbiera, Sabine Herterich, Silvia De Francia, Silvia Della Casa, Anna Calabrese, Alfredo Pontecorvi, Marcus Quinkler, Tina Kienitz, Massimo Mannelli, Letizia Canu, Anna Angelousi, Vasileios Chortis, Matthias Kroiss, Massimo Terzolo, Martin Fassnacht and Cristina L. Ronchi

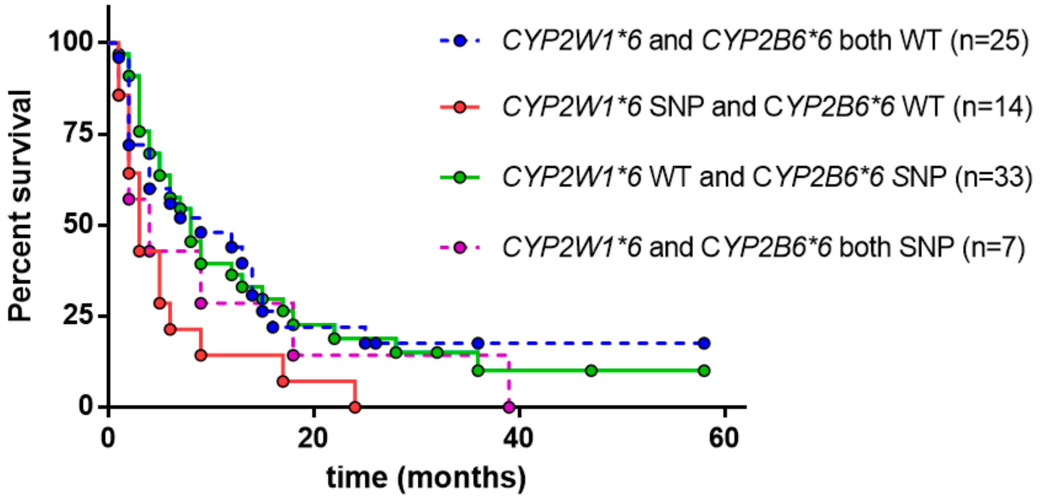

Figure S1. Combination of CYP2W1*6 and CYP2B6*6 SNP and response to mitotane therapy in group B ($n = 79$).

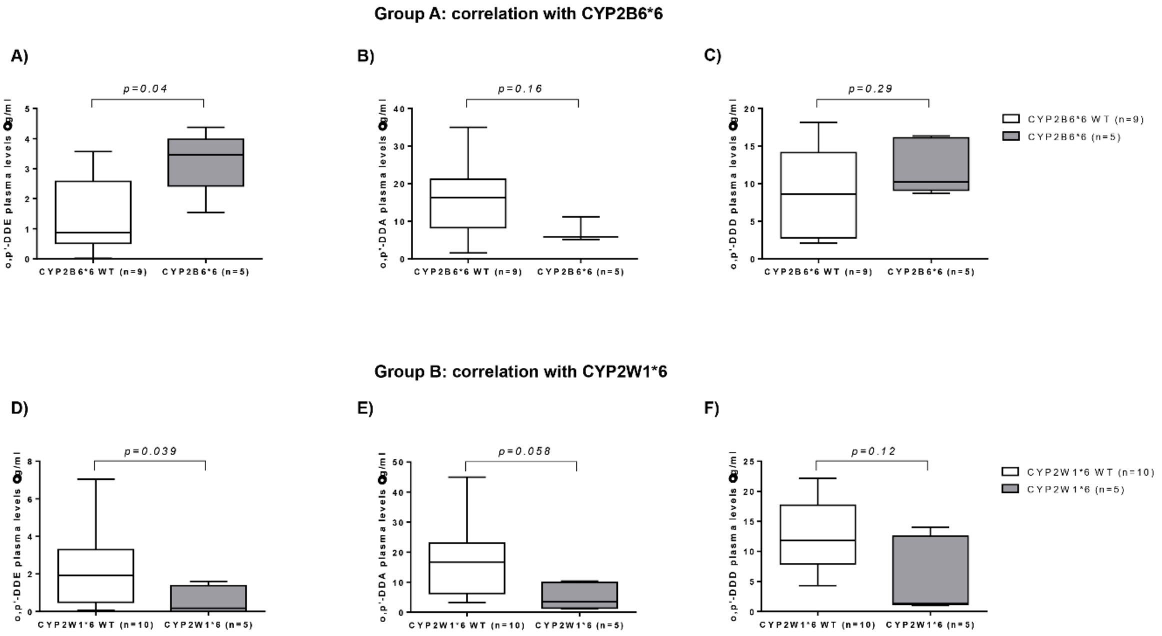

Figure S2. Correlation between mitotane metabolites measured by HPLC-UV and presence of *CYP2W1*6* and *CYP2B6*6* SNPs.

Table S1. Multivariate logistic regression analyses predicting plasma mitotane therapeutic levels.

**TTP**	**Group A**			**Group B**		
	p	**OR**	**95%CI**	***p***	**OR**	**95%CI**
Mean mitotane daily dose - gr/d	*0.18*	1.21	0.91-1.61	*0.28*	0.85	0.63-1.14
*CYP2W1*6*	*0.34*	1.58	0.61-4.11	*0.11*	0.38	0.11-1.24
*CYP2B6*6*	*0.18*	1.81	0.75-4.34	***0.04***	2.89	1.06-7.85

Group A includes patients treated adjuvantely with mitotane after radical tumor resection. Group B includes patients with not completely resected tumor, or recurrent disease not amenable to radical resection or metastatic disease at diagnosis. Mitotane therapeutic levels ≥14-20 mg/L. A *p* value in bold type denotes a significant difference ($p<0.05$). Abbreviations: Max., maximal; OR, odds ratio; TTP, time to progression; 95%CI, 95% confidence intervals.

Table S2. PCR Primers sequences used for detecting *CYP2W1* and *CYP2B6* investigated single nucleotide polymorphisms.

**Primers**	**Sequences**
CYP2W1*2	
forward	CTTCCCGCTGGCCCTACT
reverse	GGAGGCAGCTCCTCACCT
CYP2W1*5* and **6	
forward	GAGCTCTTCCTGCTGTTTGC
reverse	AGGGGAGTGGTCAGGAGGAC
***CYP2B6*6* and rs4803419**	
forward	GAGAGTTCAATGGAATTACG
reverse	AGGGAGACGGAGAGGGACA

© 2020 by the authors. Licensee MDPI, Basel, Switzerland. This article is an open access article distributed under the terms and conditions of the Creative Commons Attribution (CC BY) license (http://creativecommons.org/licenses/by/4.0/).
